# How Much Do They Know? An Analysis of the Accuracy of HIV Knowledge among Youth Affected by HIV in South Africa

**DOI:** 10.1177/2325958218822306

**Published:** 2019-01-25

**Authors:** Nicole De Wet, Joshua Akinyemi, Clifford Odimegwu

**Affiliations:** 1 Demography and Population Studies, Schools of Social Sciences and Public Health, University of the Witwatersrand, Johannesburg, South Africa; 2 Department of Epidemiology and Medical Statistics, Faculty of Public Health, College of Medicine, University of Ibadan, Ibadan, Nigeria

**Keywords:** HIV/AIDS knowledge, youth, South Africa, logistic regression

## Abstract

HIV/AIDS prevalence rates in South Africa are among the highest in the world. The key to reducing transmission is the dissemination of accurate knowledge. Here, we investigate the accuracy of HIV/AIDS knowledge among youth affected by the disease. Data from the Fourth South African National HIV, Behaviour and Health Survey (2012) are used and a weighted sample of 4 095 447 youth (15-24 years old) who have known or cared for someone with HIV/AIDS are analyzed. Results show that more than one-third (40.37%) of youth in South Africa are affected by the disease. One-quarter of the affected youth have 75% accurate knowledge of the virus, while only 10% have 100% accurate knowledge. Rural place of residence (odds ratio [OR] = 0.61) and looking for work (OR = 0.39) are less likely to have accurate knowledge. Youth without disabilities (OR = 2.46), in cohabiting (OR = 1.69), and in dating (OR = 1.70) relationships are more likely to have accurate knowledge. In conclusion, in order to reduce HIV incidence and combat HIV myths, efforts to improve the accuracy of HIV knowledge among youth affected by the disease are needed. There should be more community-based campaigns to target unemployed youth in the country.

**What do we already know about this topic?**

Youth do not have comprehensive HIV knowledge.

**How does your research contribute to the field?**

This study identifies which knowledge is largely unknown among youth affected by HIV.

**What are your research's implications toward theory, practice, or policy?**

Through identification of the exact knowledge youth affected by the disease are lacking, programmes in South Africa can make efforts to improve interventions and knowledge- based outreach.

## Introduction

Accurate knowledge of HIV/AIDS is pivotal to prevent further transmission of the disease. HIV knowledge is the correct information regarding modes of transmission, high-risk behaviors, and prevention and care strategies.^1^ The need for reliable, valid, and accurate tools for testing HIV knowledge has led to the development many different questionnaire scales over the years.^2–4^ These tools are conceptualized to evaluate the extent to which knowledge exists by age-group, sex, and other subpopulations in a number of different countries.^5–7^ These tools have also been adopted and adapted in various surveys, including population-based household and sexual behavior surveys.^8,9^

A number of studies have identified the determinants of HIV knowledge.^10–13^ One study found that women with more education and who are wealthy are more likely to know about HIV protective behaviors, including consistent condom use, and are less likely to have misconceptions about modes of transmission.^11^ Education, in particular the ability to read, makes written information, including pamphlets, billboards, newspaper articles, and health briefs and policy documents, easier to comprehend. Education also makes it possible to critically evaluate information that is disseminated and decide what correct and incorrect knowledge is. Another study of young, urban women in Kenya found that older youth (20-24 years old), having been for at least 1 HIV test in their lifetime and knowing someone, or knowing someone who died from AIDS increases the likelihood of comprehensive HIV knowledge.^12^ Older youth are more likely to have completed secondary education compared to adolescents (15-19 years old). For this reason, more education could translate into being able to comprehend more information regarding the disease. In addition, HIV-testing facilities offer trained consultation and support. Therefore, persons who have tested have access to a trained consultant or nurse to whom they may pose questions about the disease. Finally, knowing someone with the disease allows for dialogue and conversation regarding modes of transmission and protective behaviors between 2 or more individuals, one of whom would be speaking from experience.

Among youth in South Africa, the dissemination of accurate knowledge has been hindered by the perpetuation of AIDS myths and misinformation.^14–16^ Of notable importance is the impact false information has on sexual violence in the country, with 1 study reporting that 9.4% of adolescent boys, who believed that rape is a cure for HIV, demonstrated sexual violence toward a female partner.^17^ And with current youth HIV prevalence rates in the population being as high as 7.1%, there is need to reassess the accuracy of HIV knowledge among youth in the country.^18^

Further, while youth tend to have some or incomplete knowledge about HIV and how it is transmitted, evidence suggests that youth affected by the virus, that is knowing someone with HIV/AIDS in their households and/or communities, have better knowledge than youth who are not affected.^19,20^ However, even among youth affected by the disease, knowledge is not 100% accurate, yet these youth can play an important role as peer educators in preventing the spread of the disease within their households and communities, provided that they have the most accurate (100%) knowledge of HIV and AIDS. Therefore, the primary purpose of this study is to identify the factors associated with HIV knowledge accuracy among youth affected by the disease in South Africa.

## Methods

### Study Design

This is a cross-sectional study using the Fourth South African National HIV, Behaviour and Health Survey, 2012 (http://www.hsrc.ac.za/en/researchdata/).

### Survey and Sample

The 2012 survey is the fourth in the series of national household HIV surveys conducted by a consortium of scientists led by the Human Sciences Research Council.^21^ The data pertain to the HIV status, demographic, socioeconomic, and behavioral characteristics of the sample.^21^ In the 2012 survey, over 38 000 people of all ages were interviewed.^21^ The number of youth who participated in the survey is (unweighted) 8221. For this study, a weighted sample of youth affected by HIV was determined through positive responses (yes) to any of the following questions “What has made you take the problem of HIV/AIDS more seriously?”, with response options of “knowing or talking to someone with HIV/AIDS”; “Caring for a person with HIV/AIDS”; “Knowing someone who has died of AIDS.” A weighted total sample of 4 095 447 youth affected by HIV/AIDS was identified. The percentage of youth affected by HIV/AIDS is 40.37%.

### Variable Definitions

#### Dependent Variable

The dependent variable in this study is “accuracy of HIV knowledge” which is defined as having correct knowledge of all (100%) questions from the survey. Correct answers to each of the questions have been determined from similar studies that have used the same questions.^12,22^ Using 8 questions on HIV knowledge, a variable showing the percentage of correct answers was created. The survey asked a number of questions on HIV knowledge, attitudes, perceptions and behaviors (KAPB).^21^ There were only 8 questions pertaining to knowledge specifically, the rest were measurements of attitudes, perceptions, and behaviors and had been adopted from a United Nations Programme on HIV and AIDS recommended source.^23^ This study specifically focuses on the knowledge aspect of the KAPB regarding HIV and AIDS. The reason why knowledge is isolated as an outcome of this study is because knowledge can shape attitudes, perceptions, and even behaviors, particularly among youth.^24^ Knowledge therefore is a key initial component to shaping KAPB among youth affected by the disease. The questions are: (1) “Can AIDS be cured?”; (2) “Can a person reduce their risk of HIV by having fewer sexual partners?”; (3) “Can a healthy-looking person have HIV?”; (4) “Can HIV be transmitted from a mother to her unborn baby?”; (5) “Can the risk of HIV transmission be reduced by having sex with only one uninfected partner who has no other partners?”; (6) “Can a person get HIV by sharing food with someone who is infected?”; (7) “Can a person reduce the risk of getting HIV by using a condom every time he/she has sex?”; and (8)”Can medical male circumcision reduce the risk of HIV infection in males?”.

#### Independent Variables

The predictor variables for the study include age (15-19 and 20-24 years old); sex (male or female); race (African, colored, white, Indian or Asian or other); place of residence (urban or rural); self-reported disability status (yes or no); employment status (employed, not looking for work, student, looking for work, unable to work); education status (in school, not in school—completed grade 12 or not in school for another reason); marital status (married, cohabiting, dating and not living together, single or other); and source of HIV knowledge (peers or family, religious institution or community and AIDS organization, clinic or hospital). For the last variable, 11 categories of sources of information were grouped into the 3 variable responses.

### Statistical Analysis

Frequency and percentage distributions are used to show the characteristics of the sample. Cross-tabulations are done to show the distribution of the sample by independent variables, and statistical significance (*P* values) is shown. An adjusted binary logistic regression model was fitted at a 95% level of significance to identify the likelihood of accurate (100%) HIV knowledge by respondents’ characteristics. Accurate knowledge (1) in the model is measured as correct responses to all 8 questions while, 0 to 7 correct answers is considered inaccurate knowledge (0). All tests were done using STATA version 13.

## Results

**Figure 1.**
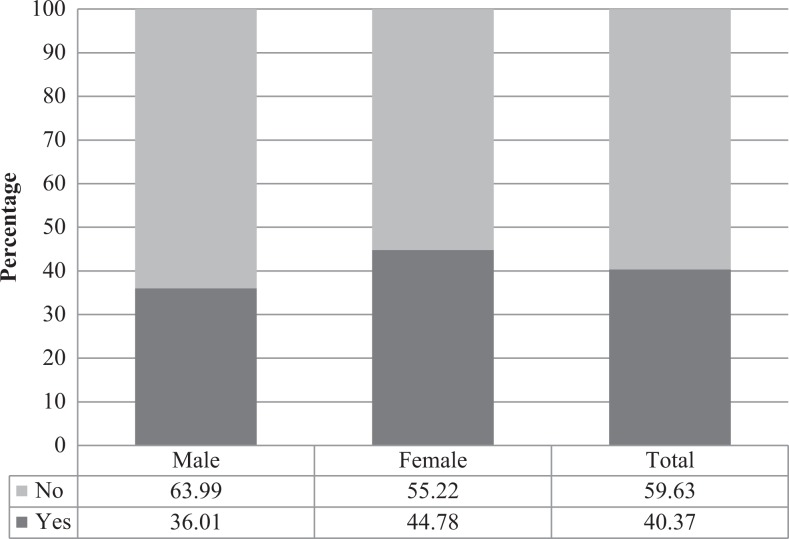
Weighted percentage distribution of youth affected (yes) and not affected (no) by HIV by sex (weighted).

Figure 1 shows that 36.01% of male and 44.78% of female youth are affected by HIV. A total of 40.37% of all youth in the country are affected by HIV.

Table 1 shows that most youth (83.35%) are aware that a person with HIV can look healthy. A further 80.67% are aware that HIV cannot be transmitted by sharing food with an HIV-positive person. However, less than half of the sample (48.13%) is aware that medical male circumcision reduces the risk of HIV infection among males.

**Table 1.** Ranking of Correct HIV Knowledge Questions Among Respondents Affected by HIV.

Knowledge of HIV questions	Correct answer	Respondents N = 4 095 447	
		N	%
Can a Healthy looking person have HIV?	Yes	3 413 468	83.35
Can a person get HIV by sharing food with someone who is positive?	No	3 303 759	80.67
Can a person reduce the risk of HIV by using a condom every time he has sex?	Yes	3 172 419	77.46
Is there a cure for HIV?	No	3 082 490	75.27
Can HIV be transmitted from a mother to her unborn child?	Yes	3 007 404	73.43
Can the risk of HIV transmission be reduced by having sex with only one partner?	Yes	2 831 754	69.14
Can a person reduce the risk of HIV by having fewer sexual partners?	Yes	2 440 196	59.58
Can medical circumcision reduce the risk of HIV infection in males?	Yes	1 971 046	48.13

**Figure 2.**
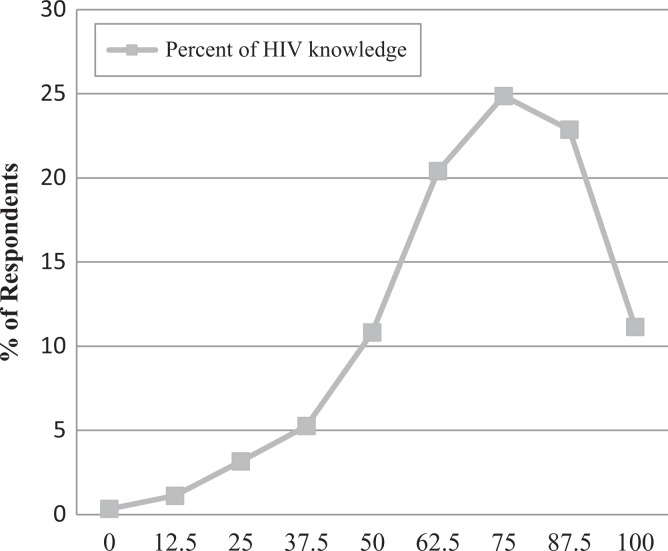
Weighted percentage distribution of accuracy of HIV knowledge (in percentages) among youth.

Figure 2 shows that only 11% of youth who are affected by HIV have 100% accuracy of HIV knowledge. Most youth, 25% of the sample, has 75% accurate knowledge. This is equivalent to getting 6 of the 8 questions correct.

Table 2 shows that the majority of youth do not have 100% accurate knowledge of HIV. Among those with 100% accuracy, most are 20 to 24 (12.32%) years old, reside in urban areas (13.48%), have no disability (11.38%), are employed (17.38%), married (13.84%), and source their information from peers and family members (19.64%). By race, the African population has the lowest percentage of complete accuracy at 10.56%, with Indian/Asian youth having the highest at 40.21%. Finally, only 10.49% of youth with 100% accuracy obtain their information from an AIDS organization or clinic.

**Table 2.** Weighted Percentage Distribution of Accuracy of HIV Knowledge (in Percentages) by Respondents’ Characteristics, Row Percentages.

Characteristics	All (N = 4 095 447)	Accuracy of HIV Knowledge			
		0%	≤50%	>50%/ <100%	100%
Age-group^a^					
15-19 (years)	41.46	0.23	23.35	66.94	9.49
20-24 (years)	58.54	0.42	18.24	69.01	12.32
Sex^a^					
Male	44.86	0.5	22.03	65.64	11.83
Female	0.00	0.22	19	70.19	10.59
Race^a^					
African	89.64	0.37	20.58	68.48	10.56
White	7.24	0	7.76	77.93	14.31
Colored	7.24	0.13	23.33	62.64	13.89
Indian/Asian	0.83	0.32	6.75	52.71	40.21
Other	0.03	0	0	86.83	13.17
Place of residence^a^					
Urban	58.99	0.07	15.81	70.63	13.48
Rural	41.01	0.74	26.9	64.58	7.79
Disability^a^					
Yes	1.40	0	34.69	63.23	2.08
No	98.46	0.35	20.08	68.18	11.38
Unsure	0.14	0	96.55	3.45	0
Employment Status^a^					
Employed	18.09	0.19	16.26	66.17	17.38
Not looking for work	30.17	0.73	20.27	69.05	9.95
Student	46.08	0.22	21.81	68.70	9.27
Looking for work	5.20	0.06	24.31	70.29	5.35
Unable to work	0.45	0	37.99	52.85	9.16
Education^a^					
In school	87.19	0.16	25.09	66.6	8.15
Not in school—Completed Grade 12	3.34	0	20.34	62.45	17.21
Not in school—other reason	9.47	0	24.47	61.53	14
Marital status^a^					
Married	3.77	0.08	6.59	79.48	13.84
Cohabiting	5.20	0	18.85	59.63	21.52
Going steady	46.34	0.22	19.51	68.13	12.14
Single	42.12	0.57	22.04	68.38	9
Other	2.57	0	27.28	65.26	7.46
Source of HIV Knowledge^a^					
Peer/family	9.51	0	18.57	61.79	19.64
Religious/community	4.28	1.02	26.87	62.77	9.34
AIDS org / clinic	86.21	0.32	20.18	69	10.49

^a^ *P* values <.05.

Table 3 shows the results of the adjusted binary logistic regression model for accuracy of HIV knowledge by characteristics of the respondents. The results show that rural place of residence (odds ratio [OR] = 0.61), looking for work (OR = 0.39), and not in school for another reason (OR = 0.89) are less likely to have accurate HIV knowledge. Alternatively, not having a disability (OR = 2.46), cohabiting (OR = 1.69), and dating (OR = 1.70) are associated with higher likelihood of accurate HIV knowledge. The results show that age, sex, race, and source of knowledge are not statistically significant predictors of accuracy of HIV knowledge.

**Table 3.** Logistic Regression Model of the Likelihood of Accurate HIV Knowledge by Respondent’s Characteristics, Showing Odds Ratios.

Characteristics	Odds Ratio	*P* Value	95% Confidence Interval	
Age-group (RC:15-19)				
20-24	0.23	.167	0.0280	1.8539
Sex (RC: Male)				
Female	1.17	.357	0.8363	1.6422
Race (RC: African)				
White	4.43	.158	0.5608	34.9257
Colored	1.06	.794	0.6860	1.6372
Indian/Asian	1.67	.375	0.5399	5.1415
Place of residence (RC: Urban)				
Rural	0.61*	.000	0.4923	0.8725
Disability (RC: Yes)				
No	2.46*	.004	1.9854	3.0104
Employment status (RC: Employed)				
Not looking for work	1.05*	.035	1.0302	3.5641
Student	0.70	.576	0.2019	2.4329
Looking for work	0.39*	.045		
Unable to work	1.46	.713	0.1941	10.9718
Education (RC: In School)				
Not in school—Completed Gr 12	1.09*	.000	1.0421	2.9326
Not in school—Other reason	0.89*	.003	0.5672	0.9236
Marital status (RC: Married)				
Cohabiting	1.69*	.026	1.4387	1.8426
Dating	1.70*	.005	1.5386	1.8924
Single	0.61	.144	0.3173	1.1826
Other	0.35	.011	0.1552	0.7876
Source of HIV Knowledge (RC: Peers/Family)				
Religious/community	1.77	.365	0.5160	6.0404
AIDS org / clinic	2.13	.043	1.0252	4.4197

*p-value < 0.05. RC = Reference Category.

## Discussion

The aim of this article is to identify the level and factors associated with accurate HIV knowledge among youth affected by the disease. More than one-third of all youth in the country are affected by HIV/AIDS. This is plausible considering HIV infection rates in the country are high at 12.7% of the total population.^25^ Also, HIV disclosure is becoming less stigmatized in the country with research showing that among HIV-positive females in the Western Cape province, disclosure of status to some family, friends, and partners was common practice, and tacit exposure, seen through taking medication in front of others, was not uncommon.^26^ This makes it more likely that youth in the country would be aware of someone living with or who has died from the disease in their households and communities.

A small percentage of youth affected by HIV/AIDS have, by this conceptualization, accurate knowledge of the disease. Another study using the same data, but on all youth, found that HIV knowledge has been decreasing over time, from 31.5% accurate knowledge in 2008 to 26.8% in 2012^18^ AIDS myths and misconceptions create skepticism and make youth less likely to believe in research-based knowledge that gets disseminated.^27–29^

While more than two-thirds of the sample are aware that an HIV-positive person can appear healthy and know that sharing food with an HIV-positive person does not spread the disease, still less than half know that medical male circumcision can reduce the risk of infection. This could be due to the recentness of the finding and the consequent debate that surrounds the generalizability of the results. At present scholars and practitioners are moot on the issue; however, the empirical consensus is appearing to skew on the side of male circumcision being a protective factor.^30–32^

More positively, youth in sexually active relationships (cohabiting, dating, and married) are more likely to have accurate knowledge than single youth. This is encouraging as it suggests that sexually active youth are aware of HIV/AIDS and that knowledge can be disseminated to their partners. However, research has shown that knowledge of HIV does not always translate into practicing protective behaviors.^33,34^ One study found that young females who have knowledge of the disease but are in physically abusive relationships are unable to negotiate condom use with their partners.^35^ Another study found that while youth know that condoms prevent the transmission of HIV, they prefer not to use them.^36^ This literature and the result from this study suggest the need to further investigate the protective behaviors of couples who have accurate HIV knowledge.

This study contributes to the existing literature first by identifying the exact knowledge that youth are lacking. From the results, there is more that needs to be done to promote male circumcision information and practices as a viable preventative strategy. Second, the study found that youth with no employment have less knowledge than employed youth. For this reason, residential community-based programs designed to target youth who do not have places of employment should be increased. With the current unemployment rate of youth being as high as 32.4%,^37^ the goal should be to disseminate HIV knowledge in areas of social interactions instead of at formal places of work and schools.

The study has a few limitations. First, accuracy of HIV knowledge is based on a few questions, which do not include injecting drug use and blood transfusion modes of transmission. A survey, including these questions, should be designed to capture more comprehensive knowledge. Second, although sources of knowledge did not prove statistically significant, this study does show a more detailed study of these sources should be conducted to identify what and how much information is disseminated by different members of the youth’s networks.

In conclusion, in order for South Africa to reduce the impact of AIDS myths and misconceptions on incidence rates, efforts to increase HIV knowledge need to be made. Youth affected by the disease in their households and communities do not have 100% HIV knowledge. However, youth affected by HIV could become peer-educators and assist in disseminating knowledge in their networks and communities. And while there is no guarantee that knowledge dissemination will directly change sexual behaviors, there is the possibility that more knowledge of HIV will work indirectly to change sexual practices through dismissing AIDS myths and encouraging evidence-based safer sex habits. For this reason, youth should receive more information through public campaigns and strategies to properly protect themselves from the disease.
